# Innovations in the Speciation of Organolead Compounds in Water: Towards a More Rational, Rapid, and Simple Analytical Process

**DOI:** 10.1100/tsw.2002.339

**Published:** 2002-05-15

**Authors:** J.R. Baena, M. Gallego, M. Valcarcel

**Affiliations:** Analytical Chemistry Division, University of Córdoba, E-14071 Córdoba, Spain

**Keywords:** organolead, speciation, fullerene, continuous flow systems

## Abstract

Speciation analysis calls for rapid, simple systems for minimizing errors made in the most troublesome of all steps in the analytical process: sample preparation. In this context, continuous-flow systems are of great help. The evolution in the different methodologies enabled solutions to the main shortcomings occurring from the lack of selectivity of using RP–C_18_ as sorbent material. One solution was a shift to more sensitive and selective, but only partially automated, systems employing C_60_ fullerene and Grignard’s reagent; another was a shift to completely automated systems employing sodium tetrapropylborate; and a final solution was to employ the simplest possible configuration by removing the reagent stream. The analytical methods developed allowed the identification and quantification of different organolead species at the pg/ml levels in rainwater samples, with precision (RSD) of about 5% and recoveries ranging from 92 to 100%.

## INTRODUCTION

Organolead compounds are released into the atmosphere by direct evaporation from gasoline during transport and storage or as a consequence of the incomplete combustion of the leaded gasoline in explosion motors. Once in the atmosphere, volatile tetraalkyllead compounds or particle-adsorbed ionic alkyllead compounds can be transported long distances until they are dissolved by the rain and sent back to the ground, where they reach superficial and groundwaters.

The Achilles' heel of the conventional method used to implement speciation of organolead compounds using GC/MS is the sample preparation, which is a multistep procedure involving measurement of sample volume, addition of complexing reagents, liquid or solid extraction into organic solvents, filtration, derivatization, destruction of the excess of derivatizing reagent, and final injection into the GC/MS. These steps are highly time consuming, require the full participation of the analyst, and are the source of both systematic and random errors as well as source of risks for the analyst as a consequence of handling instable and toxic compounds. The main aim of this work is to minimize or avoid these drawbacks by developing a series of innovations; the most relevant of these are as follows:

- Use of $C_{60}$ fullerene and derivatives as sorbents for preconcentration and clean-up;
- Automation of preliminary operations such as sample volume measurement, filtration, dual derivatization, etc.; and
- Implementation of a simple and rapid sample screening system to avoid the systematic use of sophisticated instrumentation in routine laboratories.

A series of automatic methods have been developed by improving performance characteristics such as simplicity, reduction of human participation, rapidity, selectivity, and sensitivity. The sequential improvement has been achieved by implementing the following changes:

- The use of different sorbent materials (RP–$C_{18}$, $C_{60}$ fullerene, and its diethyldithiocarbamate derivative, $C_{60}$-DDC);
- The substitution of the manual derivatization procedure of the analytes by its implementation in a continuous flow system;
- The replacement of Grignard reagents imposing the use of an argon atmosphere by $NaBPr_4$, which simplifies the handling of the sample;
- The covalent immobilization of sodium diethylditihiocarbamate on $C_{60}$ fullerene; and
- The simplification of complex flow systems involving several peristaltic pumps, valves, and flow lines to achieve simpler systems with a higher ruggedness and reliability.

In this communication the different automatic manifolds developed and the corresponding figures of merit are presented and critically discussed, including the limitations and challenges in this context.

## CHEMICALS AND INSTRUMENTATION

Standard solutions (500 mg/ml) of trimethyllead ($TML^+$) and triethyllead ($TEL^+$) chloride (Alfa, Barcelona, Spain) were prepared by dissolving the proper amounts of the salts in ultrapure (Milli-Q) water. Dimethyllead ($DML^{2+}$) and diethyllead ($DEL^{2+}$) solutions (25 mg/l) were prepared from $TML^+$ and $TEL^+$ standards through a reaction with 0.1 M iodine monochloride (ICl). Sodium diethyldithiocarbamte (NaDDC) (Sigma-Aldrich, Madrid, Spain) was dissolved in ultrapure water up to a concentration of $3 \times 10^{-3}$ M.

Preconcentration of the analytes was performed using a continuous-flow system consisting of two injection valves (Rheodyne, 5041, Cotati, CA), a Gilson peristaltic pump (Minipulse 2, Villiers-le-Bel, France), and PTFE tubings for connections. The sorbent materials (RP–$C_{18}$, $C_{60}$, and $C_{60}$-NaDDC derivative) were placed into minicolumns constructed from PTFE with small cotton wools in both ends to prevent loss of materials. Propylmagnesium chloride (PrMgCl) (Sigma, Madrid, Spain) in a concentration of 2.0 M in diethyl ether, and sodium tetrapropylborate ($NaBPr_4$) (Galab, Geesthacht, Germany) in a concentration of 1.2 M in ethanol were employed as derivatising reagents.

Detection of organolead compounds was accomplished by using a Fisons GC8000/MD800 GC/MS from ThermoQuest (Madrid, Spain) with electronic pressure control and governed via MASSLAB software (also from ThermoQuest). Separation of the analytes was achieved in a capillary column (30 m × 0.25 mm i.d., 0.25 μm) HP-5-MS (Supelco, Madrid, Spain) using ultrapure grade helium (6.0, Air Liquide, Seville, Spain) at a constant flow-rate of 0.9 ml/min. The GC injection port, GC/MS interface, and MS source temperatures were kept at 250, 250, and 200ºC, respectively. The column temperature was initially set at 50ºC for 1 min and then raised at 30ºC/min to 250ºC, where it was held for 1.5 min. A 1-μl aliquot of the sample was injected in the split mode (1:25). Mass spectra were obtained in the electron impact ionisation mode at 70 eV, and the mass spectrometer was operated in the selective ion monitoring (SIM or SIR) mode, with the characteristic ions selected for identification and quantitation (underlined) of each analyte being as follows: $TML^+$ 208, <u>253</u>, 281; $DML^{2+}$ 208, <u>281</u>, 309; $TEL^+$ 208, 237, <u>309</u>; $DEL^{2+}$ 208, <u>251</u>, 322; $Pb^{2+}$ 208, <u>251</u>, 337.

## SPECIATION OF ORGANOLEAD COMPOUNDS

Continuous-flow systems are a very useful tool to employ in analytical chemistry, especially when the manual procedures are based on sequential steps that are time consuming and require the complete attention of a qualified operator. Their use enhances the sensitivity and precision of the method and at the same time reduces the reagent consumption and minimizes the risks of contamination of the samples or loss of the analytes.

In a first attempt to automate the speciation of organolead compounds, a RP–$C_{18}$ column has been employed following the procedure described elsewhere[1] and schematized in Fig. 1. Sample volumes of 5 to 25 ml containing organolead compounds were aspirated into the flow system (flow rate, 2.2 ml/min) and merged with a $3 \times 10^{-3}$ M NaDDC stream (flow rate, 0.4 ml/min) in order to form a neutral chelate that is later retained onto the RP–$C_{18}$-sorbent column. Other components of the sample are driven to waste, so that this sorption is also an effective clean-up step. Once the analytes have been adsorbed onto the column, a small volume (200 μl) of *n*-hexane is introduced into the system by means of the second injection valve, and lead to the column by an air carrier stream (flow rate, 4.5 ml/min), thus enabling the elution of the chelates. The eluate is collected in a vessel and subjected to the required derivatization step with PrMgCl in order to render the ionic alkyllead compounds into the more volatile and thermally stable tetraalkyllead species, which are suitable for injection into the GC/MS. The system allowed the determination of $TML^+$, $TEL^+$, $DML^{2+}$, and $DEL^{2+}$ in concentrations commonly found in real-world samples; however, the presence of other metallic ions such as $Zn^{2+}$, $Fe^{3+}$, $Cu^{2+}$, $Mn^{2+}$, or $Co^{2+}$ in concentration ratios from 300 to 700 relative to the lead content in the sample was found to interfere in the determination of organolead compounds by displacing the lead chelates from the active sites of the surface of the sorbent.

This lack of selectivity can be overcome by using $C_{60}$ fullerene as sorbent material instead of RP–$C_{18}$. The unique electronic characteristics of this material provide a special preference for π–electron donor solutes to be adsorbed on the fullerene surface, vs. charged or nondonor species; thus, the inclusion of a $C_{60}$ minicolumn in the configuration of the continuous-flow system enhances the selectivity of the method, with $Fe^{3+}$ showing the only observed interference which can be easily avoided by adding 1 ml of glacial acetic acid per litre of sample as demonstrated elsewhere[2].

Despite the effectiveness achieved in the preconcentration of organolead species, their derivatization was still a tedious manual task. Grignard reagents are air- and moisture-sensitive,

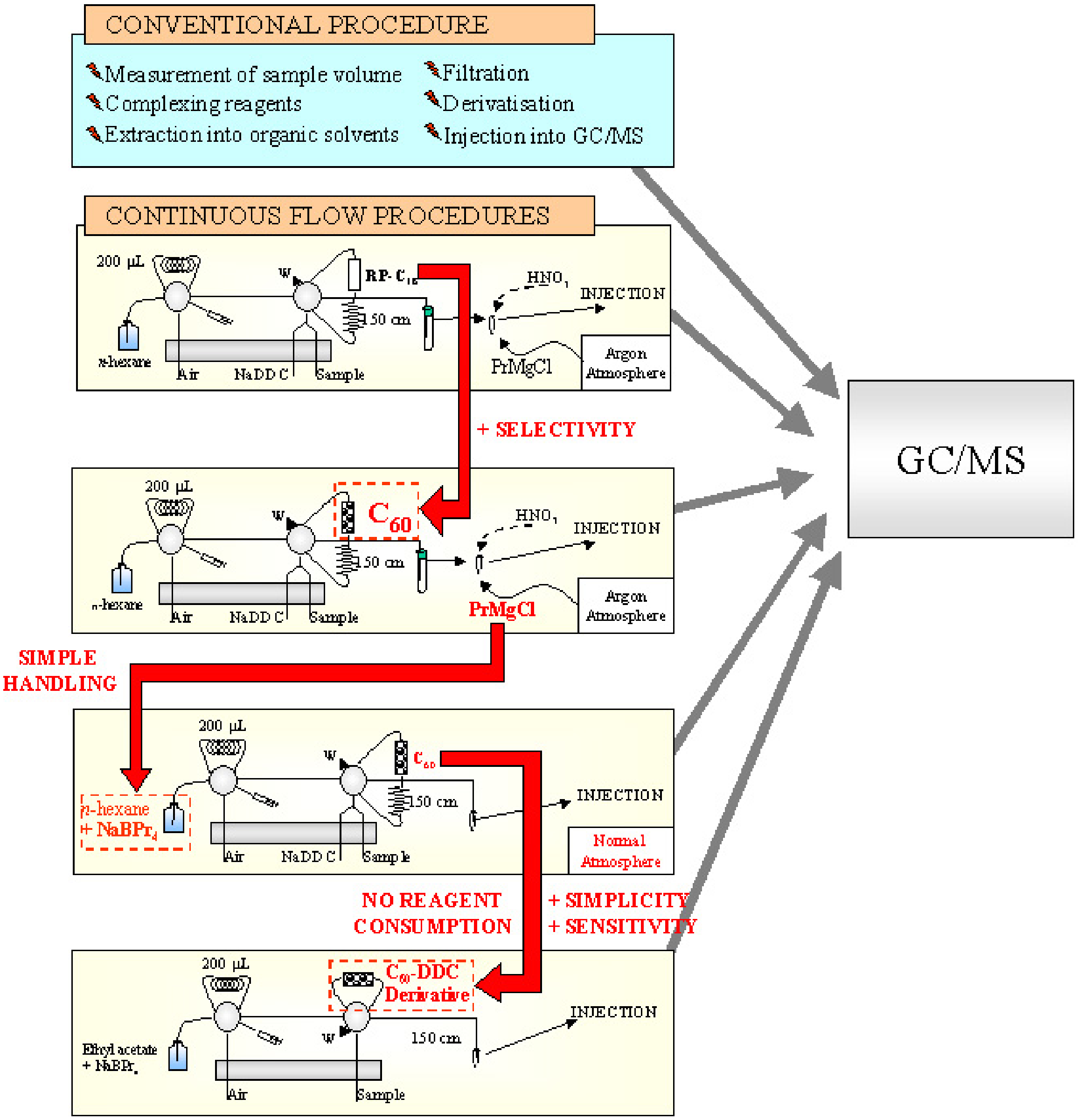

**FIGURE 1.** Evolution of the continuous-flow systems employed for the speciation analysis of organolead compounds in rainwater samples by GC/MS.

and their handling usually involves the use of an inert atmosphere. Hence, vials with the proper amount (70 µl) of PrMgCl must be filled and sealed with a septum cap in order to make possible the following addition of the eluate (*n*-hexane containing the preconcentrated analytes). After the derivatization time (8 min), the excess of the Grignard reagent must be destroyed by the addition of 0.5 ml of water. The organic phase can then be separated from the aqueous phase, and any residual water must be removed from the extract by adding anhydrous sodium sulphate prior to the final injection into the GC/MS. Limits of detection between 4 and 15 ng/l of the alkyllead compounds (expressed as lead) are achieved employing 5 ml of sample volume.

**TABLE 1**
**Analytical Characteristics for Speciation Analysis of Organolead Compounds in Rainwater Samples Using a $C_{60}$-DDC Derivative as Sorbent Material**

| | $TML^+$ | $DML^{2+}$ | $TEL^+$ | $DEL^{2+}$ |
|---|---|---|---|---|
| Detection limit (pg/ml as Pb) | 5 | 15 | 4 | 10 |
| Linear range (ng/ml as Pb) | 0.02–4 | 0.04–4 | 0.02–3 | 0.03–4 |
| RSD (%) n = 11, 0.5 ng/ml as Pb | 5.5 | 4.6 | 4.8 | 5.8 |
| Recoveries (%) n = 5, 0.1 ng/ml as Pb | 92–96 | 95–99 | 95–99 | 94–100 |

*Note*: Sample volume = 5 ml; data taken from[6].

As has been mentioned, the procedure based on the employment of a Grignard reagent is highly tedious and time consuming. For this reason, a number of alternative derivatizing reagents such as tetraalkylborates (tetrabutylammonium tetrabutylborate[3] and sodium tetrapropylborate[4]) have arisen in the last years. These compounds possess acceptable stability in solution and can be handled in open atmosphere without special cares, in contrast to Grignard reagents. In addition, the recent commercial availability of sodium tetrapropylborate ($NaBPr_4$) contributes to its wide use in organometallic speciation. Actually, $NaBPr_4$ has been used in a continuous-flow system in order to enable the complete automation of the speciation procedure for organolead compounds[5]. In this method, the new derivatizing reagent is included in the eluent in a concentration of $2.4 \times 10^{-3}$ M as the solution introduced into the flow system. The elution of the analytes also implies the beginning of the derivatization, which can be completed in only 3 min. After that time, the eluate can be directly injected into the GC/MS, providing detection limits from 4 to 12 ng/l for a sample volume of 5 ml.

The final improvement of the flow system consists of the substitution of the $C_{60}$ sorbent column for the new $C_{60}$ derivative, which includes a diethyldithiocarbamate moiety in the molecule. The synthesis, characterization, and optimization of the method are clearly described elsewhere[6]. In this case, the method is simplified since no addition of the reagent is needed owing to the fact that the neutral chelates are formed *in situ* in the $C_{60}$ derivative (see Fig. 1). Both elution and derivatization steps are accomplished simultaneously. Sensitivity as well as simplicity of the system are enhanced by use of this new approach. The main analytical features of the method for a sample volume of 5 ml are shown in Table 1. Samples collected in different locations in Spain revealed the presence of all lead species except $TML^+$, probably as a result of environmental degradation to $DML^{2+}$ and $Pb^{2+}$. All other species were detected in concentrations ranging from 10 to 90 pg/ml for $DML^{2+}$, from 30 to 150 pg/ml for $TEL^+$, and from 36 to 100 pg/ml for $DEL^{2+}$[1,5,6].

## CONCLUSIONS

The preliminary operations of the speciation analytical process have been substantially simplified and automated by using specially designed continuous-flow systems to perform the treatment and derivatization operations involved. In this context, fullerene and derivatives have been shown to be suitable sorbent materials for environmental speciation, with substantial advantages over classical sorbents such as silica-bonded $C_{18}$ materials. The proposed methodologies allowed the direct discrimination of rainwater from different locations in Spain.

## BIOSKETCHES

**Josefa R. Baena** is Researcher in the Analytical Chemistry department at the University of Córdoba, Spain. She finished her Ph.D. thesis on analytical applications of fullerenes, studying their potential as sorbent materials in continuous flow systems to enable the preconcentration of organometallic compounds in environmental samples.

**Mercedes Gallego** is Titular Professor of Analytical Chemistry at the University of Córdoba, Spain. Her research interests cover most aspects of automatic sample preparation for environmental, food and clinic samples, combined with gas chromatography and atomic absorption spectrometry techniques.

**Miguel Valcárcel** is Professor of Analytical Chemistry at the University of Córdoba, Spain. He is Head of the research group on automation, simplification and quality of (bio)chemical measurement processes. He is Chairman of the Analytical Division of the Federation of European Chemical Societies.
